# Asymptomatic Hyperuricemia as an Independent Risk Factor for Myocardial Infarction in Adult Population: A Four-Year Follow-Up Study

**DOI:** 10.7759/cureus.34614

**Published:** 2023-02-03

**Authors:** Abdul Subhan Talpur, Abdul Fattah, Hewad Hewadmal, Farukhzad Hafizyar, Jawad Farooq, Tanveer Ahamad Shaik, Laila Tul Qadar, Syed Muhammad Hussain Zaidi, Sarmad Pirzada, Abdul Rasheed Bahar

**Affiliations:** 1 Medicine, Liaquat University of Medical and Health Sciences, Jamshoro, PAK; 2 Internal Medicine, Indus Medical College, Tando Muhammad Khan, PAK; 3 Cardiology, Sheikh Zayed Medical College/Hospital, Rahim Yar Khan, PAK; 4 Internal Medicine, Liaquatian Research Council, Hyderabad, PAK; 5 Cardiovascular Medicine, University of Louisville School of Medicine, Louisville, USA; 6 Cardiovascular Medicine, Liaquatian Research Council, Hyderabad, PAK; 7 Internal Medicine, Dow University of Health Sciences, Civil Hospital Karachi, Karachi, PAK; 8 Internal Medicine, Dow University of Health Sciences, Karachi, PAK; 9 Internal Medicine, Trinity Health Livonia, Livonia, USA

**Keywords:** acute myocardial infarction in pakistan, cardiac risk factors and prevention, fatal myocardial infarction, non-fatal myocardial infarction, elevated uric acid levels

## Abstract

Introduction

A condition in which uric acid levels are elevated but there are no accompanying symptoms is known as asymptomatic hyperuricemia. As a result of the disparity in opinions and findings between the studies, the guidelines regarding whether or not asymptomatic hyperuricemia should be treated are unclear.

Material and methods

Between the months of January 2017 and June 2022, this research was carried out in the community in collaboration with the internal medicine unit and the public health unit of Liaquat University of Medical and Health Sciences. After obtaining informed consent from each participant, the researchers enrolled 1,500 patients in the study who had uric acid levels that were greater than 7.0 mg/dL. These patients ranged in age from 40 to 70 years old and were of either gender. As a control group, 1,500 patients were recruited who did not have abnormally high levels of uric acid. Patients were monitored for a total of 48 months or until the occurrence of a major cardiovascular event (MACCE) or death from all causes, whichever occurred first. Death, cardiovascular mortality, non-fatal myocardial infarction, and non-fatal stroke were the four categories that made up the primary outcome, also known as MACCEs.

Results

In the hyperuricemic group, the incidence of myocardial infarction that did not result in death was significantly higher than in the non-hyperuricemic group (1.6% vs. 0.7%; p-value, 0.04). However, the result was not significant for deaths from all causes, deaths from cardiovascular disease, or strokes that did not result in death.

Conclusion

Asymptomatic hyperuricemia is a potential threat to one's health that can lead to cardiovascular diseases and may go undiagnosed in some cases. It is important to remember that hyperuricemia can lead to delirious complications, so efforts should be made to perform routine monitoring and management of the condition.

## Introduction

When measured in a laboratory, hyperuricemia is indicated when the level of uric acid in the serum is greater than 7.0 mg/dL [1,2]. Increased levels of uric acid have been shown to be linked to a number of comorbid conditions, including obesity, hypertension, dyslipidemia, and diabetes, all of which have the potential to result in cardiovascular disease by a number of research studies [3-5].

Both the Rotterdam Study and the Apolipoprotein Mortality Risk study (AMORIS) found that people with high serum uric acid levels had a significantly increased risk of having a myocardial infarction [6]. According to the findings of the Losartan Intervention for Endpoint Reduction in hypertension (LIFE) study [7], the association between hyperuricemia and myocardial infarction is stronger in women than it is in men. It was demonstrated in the Mudanjiang Chronic Non-communicable Diseases Study [8] that different trajectories of serum uric acid are uniquely correlated with the risk of hypertension in middle-aged adults. According to the findings of K.C. Sung and colleagues' research carried out in Korea, hyperuricemia ought to be regarded as an independent risk factor for developing hypertension [9].

In spite of the fact that numerous regional studies have been carried out to investigate the impact that hyperuricemia has on cardiovascular outcomes, each of these studies was carried out by a single institute and involved patient populations that were at risk for developing cardiovascular diseases. In this study, we will determine the risk of cardiovascular adverse events that can be caused by asymptomatic hyperuricemia in adult patients who do not have any traditional risk factors associated with cardiovascular disease.

## Materials and methods

Between the months of January 2017 and June 2022, this research was carried out in the community in collaboration with the internal medicine unit and the public health unit of Liaquat University of Medical and Health Sciences. After obtaining informed consent from each participant, the researchers enrolled 1,500 patients in the study who had uric acid levels that were greater than 7.0 mg/dL. These patients ranged in age from 40 to 70 years old and were of either gender. Patients were selected for this study using a method called consecutive convenience non-probability sampling from a number of different screening camps. The UASure Blood Uric Acid Monitoring handheld device was used to conduct the analysis in order to determine the uric acid levels. As a control group, 1,500 patients were recruited who did not have abnormally high levels of uric acid. Participants had a smoking history, a hypertension diagnosis, a diabetes diagnosis, or a body mass index that was greater than 25 kg/m^2^. Approval on an ethical level was received from the institutional ethical review board (LUMHS/IRB/2017/01-03).

In the self-structured questionnaire, patient characteristics such as age, gender, history of smoking, blood pressure, previous history of myocardial infarction, and family history of myocardial infarction were recorded. Patients were monitored for a total of 48 months or until the occurrence of a major cardiovascular event (MACCE) or death from all causes, whichever occurred first. Death, cardiovascular mortality, non-fatal myocardial infarction, and non-fatal stroke were the four categories that made up the primary outcome, also known as MACCEs.

Patients were given instructions to follow up at community clinics once every six months, where they would also have their uric acid levels rechecked. Patients in hyperuricemic groups who had two consecutive normal readings were not considered for inclusion in the study. Patients in groups other than those with hyperuricemia who had two elevated readings were treated in the same manner and excluded from the study. Patients who did not show up for their appointments at the clinic were first contacted by phone and then had a house visit performed on them. A form requesting informed consent was filled out, and consent was obtained for this.

One hundred and forty-two (142) participants in the hyperuricemic group were unable to be followed up with, while 111 participants in the non-hyperuricemic group were unable to be followed up with. The final analysis only included information from participants who had finished the entire study. The statistical analysis was carried out with the most recent release of the statistical package for the social sciences (SPSS) (IBM Corporation, Armonk, NY, United States). While descriptive statistics were used to analyze continuous variables, which were then presented as the mean and the standard deviation (SD), frequency distributions and percentages were used to present categorical variables. The 95% confidence interval was used in the calculation of the relative risk (RR), which was done using an online calculator (medCalc). If the P value was lower than 0.05, it indicated that there was a significant difference between the two groups, thus invalidating the null hypothesis.

## Results

There was a total of 2,747 people who took part in the research for this study. The hyperuricemic group had a mean age of 47 years and 8 months, while the non-hyperuricemic group had a mean age of 46 years and 9 months; however, there was no significant difference between the two groups. In the group that did not have hyperuricemia, the body mass index was significantly higher than the group that did have hyperuricemia (22.6 0.9 vs. 22.2 1.2; p-value, 0.0001) (Table 1).

**Table 1:** Characteristics pattern among hyperuricemic vs non-hyperuricemic population. Abbreviation: SD, standard deviation. NS, non-significant. kg, Kilogram. M, metre

Characteristics	Hyperuricemic Group (n=1,358)	Non-hyperuricemia Group (n=1,389)	P-value
Age in year (Mean ±SD)	47 ± 08	46 ± 09	0.021
Uric acid in mg/dl (Mean ±SD)	7.6 ± 1.2	5.3 ± 0.8	< 0.0001
Male (%)	701 (51.6%)	689 (49.6%)	0.29
Body Mass Index (kg/m^2^)	22.2 ± 1.2	22.6 ± 0.9	< 0.0001

In the hyperuricemic group, the incidence of myocardial infarction that did not result in death was significantly higher than in the non-hyperuricemic group (1.6% vs. 0.7%; p-value, 0.04). However, the result was not significant (Table 2) when looking at mortality from all causes, mortality from cardiovascular disease, or non-fatal stroke.

**Table 2:** All causes of mortality and major cardiovascular events among the hyperuricemic vs non-hyperuricemic group.

All-cause mortality and major cardiovascular events (MACCEs)	Hyperuricemic Group (n=1,358)	Non-hyperuricemia Group (n=1,389)	P-value
All-Cause Mortality	41 (3.0%)	31 (2.2%)	0.19
Cardiovascular Mortality	14 (1.0%)	06 (0.4%)	0.06
Non-Fatal Myocardial Infarction	22 (1.6%)	11 (0.7%)	0.04
Non-Fatal Stroke	5 (0.3%)	2 (0.1%)	0.24

There was no significant difference between males and females in terms of total case mortality or major cardiovascular events that occurred in hyperuricemic groups (Table 3).

**Table 3:** All causes of mortality and major cardiovascular events in hyperuricemic groups between males and females.

All-cause mortality and major cardiovascular events in hyperuricemic group (MACCEs)	Male (n=701)	Female (n=657)	P-value
All-Cause Mortality (n=49)	26 (3.7%)	23 (3.5%)	0.83
Cardiovascular Mortality (n=20)	9 (1.2%)	11 (1.6%)	0.81
Non-Fatal Myocardial Infarction (n=32)	17 (2.4%)	15 (2.2%)	0.86
Non-Fatal Stroke (n=5)	2 (0.2%)	3 (0.4%)	0.60

## Discussion

According to the findings of our research, asymptomatic individuals with hyperuricemia have an increased risk of nonfatal myocardial infarction, even in the absence of traditional risk factors such as smoking, hypertension, diabetes, and so on [10]. Wu et al. found that asymptomatic hyperuricemic subjects who did not have any comorbidities had a significantly increased risk of developing incident coronary artery disease events that were 1.82 times higher than normal. Numerous studies have been conducted to investigate the role that uric acid plays as an independent risk factor. Uric acid has been linked as a contributor to the development of cardiovascular disease in a number of studies [11-13]. On the other hand, a number of studies have disproved the claim and have come to the conclusion that there is no connection between uric acid and cardiovascular diseases [14,15]. Because of the disparity in opinions and findings between studies, there is currently uncertainty and confusion regarding whether or not asymptomatic hyperuricemia should be treated [16]. In contrast to the findings of Wu, which found that females had a higher risk compared to males [10], our research discovered that there was no difference in the risk associated with increased uric acid between males and females.

This finding suggests that a causal link to the development of hypertension is a plausible explanation for the possible increased CAD risk in patients with hyperuricemia [17]. Second, the presence of hyperuricemia may contribute to lipid peroxidation and promote the oxidation of low-density lipoprotein cholesterol, both of which may play a role in the development of atherosclerosis and would also explain its association with CAD events [18-20]. This would explain why hyperuricemia is associated with an increased risk of cardiovascular disease. It is important to note that human atherosclerosis plaques contain a higher concentration of uric acid than do the walls of normal artery, which suggests that SUA may play a direct role in the development of atherosclerosis [21]. Thirdly, hyperuricemia may induce endothelial dysfunction, which is predicted to promote the early development of atherosclerosis and come before the formation of plaque [22]. This prediction is based on the fact that hyperuricemia is associated with an increased risk of death from cardiovascular disease.

There is a significant problem with hyperuricemia in Pakistan. Despite the fact that symptomatic and asymptomatic hyperuricemic patients were included in this study, the overall prevalence of hyperuricemia was determined to be 30.1% [23]. This is the first large local study that, to the best of our knowledge, investigates the association between asymptomatic hyperuricemia and cardiovascular risk factors by following the participants for an extended period of time. Nonetheless, this study is not without its share of restrictions and caveats. To begin, the research only removed a small number of potentially confusing factors like smoking, hypertension, diabetes, and obesity from consideration. Other potentially confusing factors, like age, stress, and hypercholesterolemia, were not excluded from the analysis. It was not studied whether treating asymptomatic hyperuricemia had any impact.

## Conclusions

Asymptomatic hyperuricemia is a potential threat to one's health that can lead to cardiovascular diseases and may go undiagnosed in some cases. It is important to remember that hyperuricemia can lead to delirious complications, so efforts should be made to perform routine monitoring and management of the condition. To better inform their decisions regarding the treatment and management of asymptomatic hyperuricemia, medical professionals should collect and analyse more robust evidence.

## References

[1] Kubota M (2019). Hyperuricemia in children and adolescents: present knowledge and future directions. J Nutr Metab.

[2] Neogi T (2011). Gout. N Engl J Med.

[3] Wilcox WD (1996). Abnormal serum uric acid levels in children. J Pediatr.

[4] Zheng R, Chen C, Yang T, Chen Q, Lu R, Mao Y (2017). Serum uric acid levels and the risk of obesity: a longitudinal population-based epidemiological study. Clin Lab.

[5] Wang Y, Hu JW, Lv YB (2017). The role of uric acid in hypertension of adolescents, prehypertension and salt sensitivity of blood pressure. Med Sci Monit.

[6] Holme I, Aastveit AH, Hammar N, Jungner I, Walldius G (2009). Uric acid and risk of myocardial infarction, stroke and congestive heart failure in 417,734 men and women in the Apolipoprotein MOrtality RISk study (AMORIS). J Intern Med.

[7] Høieggen A, Alderman MH, Kjeldsen SE (2004). The impact of serum uric acid on cardiovascular outcomes in the LIFE study. Kidney Int.

[8] Ma H, Wang X, Guo X, Li X, Qi L, Li Y (2019). Distinct uric acid trajectories are associated with different risks of incident hypertension in middle-aged adults. Mayo Clin Proc.

[9] Sung KC, Byrne CD, Ryu S (2017). Baseline and change in uric acid concentration over time are associated with incident hypertension in large Korean cohort. Am J Hypertens.

[10] Wu J, Lei G, Wang X (2017). Asymptomatic hyperuricemia and coronary artery disease in elderly patients without comorbidities. Oncotarget.

[11] Liese AD, Hense HW, Löwel H, Döring A, Tietze M, Keil U (1999). Association of serum uric acid with all-cause and cardiovascular disease mortality and incident myocardial infarction in the MONICA Augsburg cohort. World Health Organization Monitoring Trends and Determinants in Cardiovascular Diseases. Epidemiology.

[12] Weir CJ, Muir SW, Walters MR, Lees KR (2003). Serum urate as an independent predictor of poor outcome and future vascular events after acute stroke. Stroke.

[13] Bos MJ, Koudstaal PJ, Hofman A, Witteman JC, Breteler MM (2006). Uric acid is a risk factor for myocardial infarction and stroke: the Rotterdam study. Stroke.

[14] Burgos J, Cassis S, Kunstmann G, Hernández A (1991). Adrenal hemorrhage in newborn infants (Article in German). Rev Chil Pediatr.

[15] Sakata K, Hashimoto T, Ueshima H, Okayama A (2001). Absence of an association between serum uric acid and mortality from cardiovascular disease: NIPPON DATA 80, 1980-1994. National Integrated Projects for Prospective Observation of Non-communicable Diseases and its Trend in the Aged. Eur J Epidemiol.

[16] Brucato A, Cianci F, Carnovale C (2020). Management of hyperuricemia in asymptomatic patients: a critical appraisal. Eur J Intern Med.

[17] Baker JF, Krishnan E, Chen L, Schumacher HR (2005). Serum uric acid and cardiovascular disease: recent developments, and where do they leave us?. Am J Med.

[18] De Scheerder IK, van de Kraay AM, Lamers JM, Koster JF, de Jong JW, Serruys PW (1991). Myocardial malondialdehyde and uric acid release after short-lasting coronary occlusions during coronary angioplasty: potential mechanisms for free radical generation. Am J Cardiol.

[19] Iribarren C, Folsom AR, Eckfeldt JH, McGovern PG, Nieto FJ (1996). Correlates of uric acid and its association with asymptomatic carotid atherosclerosis: the ARIC Study. Atherosclerosis Risk in Communities. Ann Epidemiol.

[20] Gómez M, Vila J, Elosua R (2014). Relationship of lipid oxidation with subclinical atherosclerosis and 10-year coronary events in general population. Atherosclerosis.

[21] Suarna C, Dean RT, May J, Stocker R (1995). Human atherosclerotic plaque contains both oxidized lipids and relatively large amounts of alpha-tocopherol and ascorbate. Arterioscler Thromb Vasc Biol.

[22] Higgins P, Dawson J, Lees KR, McArthur K, Quinn TJ, Walters MR (2012). Xanthine oxidase inhibition for the treatment of cardiovascular disease: a systematic review and meta-analysis. Cardiovasc Ther.

[23] Raja S, Kumar A, Aahooja RD, Thakuria U, Ochani S, Shaukat F (2019). Frequency of hyperuricemia and its risk factors in the adult population. Cureus.

